# Sequencing and analyzing complete mitochondrial DNA of *Muscina angustifrons* (Insecta, Diptera, Muscidae)

**DOI:** 10.1080/23802359.2017.1289352

**Published:** 2017-02-21

**Authors:** Mustafa Zafer Karagozlu, Seong Hwan Park, Sang-Eon Shin, Chang-Bae Kim

**Affiliations:** a Department of Life Science, Sangmyung University, Seoul, Republic of Korea;; b Department of Legal Medicine, Korea University College of Medicine, Seoul, Republic of Korea

**Keywords:** Insecta, Diptera, Muscidae, complete mitochondrial genome, *Muscina angustifrons*

## Abstract

In this study, the complete mitochondrial genome sequenced and analyzed from a fly, *Muscina angustifrons* which collected from South Korea. The size of mitochondrial genome is 16,316 bp with 40.9% A, 12.3% C, 8.4% G and 38.4% T distribution. Furthermore, phylogenetic relationships of the superfamily Muscoidea evaluated due to mitochondrial protein coding genes. The results showed that the family Muscidae is a paraphyletic group and the closest species to *M. angustifrons* is *M. levida.* This is the third complete mitochondrial genome for the genus *Muscina* and the first genus record from South Korea.

*Muscina* is a fly genus that belongs to the family Muscidae. They are represented with 27 species and abundant in worldwide (De Carvalho et al. 2005). Species of *Muscina* on the corpses are critically important for forensic investigation for determination of post-mortem intervals and estimation of the time of death (Byrd & Castner 2010). Therefore, correct identification of the insect species from crime scenes is crucial for forensic investigations. There are only two mitochondrial genome records from *Muscina* species in the GenBank which are *M. stabulans* (KM676394) from China and *M. levida* (KT272866) from U.S.A (Lan et al. 2015; Junqueira et al. 2016). In this study we are providing the complete mitochondrial genome of *Muscina angustifrons.* This is the third complete mitogenome belonging to the genus *Muscina.*

The specimens examined in the study have been collected from shade, forest, Mt. Gaewun, Seoul/37°35′43.0”N 129°01′43.1”E, August 2016. The collected specimens deposited in Department of Legal Medicine, Korea University (16An27). DNA preparation, DNA sequencing, mitochondrial gene annotation and phylogeny reconstruction methods described in our previous study (Karagozlu et al. 2016).

The mitochondrial genome of *M. angustifrons* (GenBank Accession number: KY495724) has typical gene order and gene orientation with ancestral insect genome (Cameron 2014). It consist of 13 protein-coding genes, two rRNA genes, 22 tRNA genes and one putative control region likewise the other *Muscina* records (Lan et al. 2015; Junqueira et al. 2016). Among them *M. angustifrons* has the longest mitochondrial genome with 16,316 bp length. The reason of this difference may be the length of the putative control region because it has the longest putative control region (1369 bp) which located between 12S rRNA gene and tRNA-Ile. *Muscina angustifrons* consists of seven overlapping regions in the genome with 1 to 40 bp lengths. The largest overlapping region is located between tRNA-Leu and 16S rRNA gene. The mitogenome shows 26 intergenic sequences varying from 1 to 110 bp in lengths. The longest intergenic sequence is between tRNA-Glu and tRNA-Phe.

Furthermore phylogenetic relationship of *M. angustifrons* in the superfamily Muscoidea was investigated (Figure 1). The phylogenetic tree showed that the closest species to *M. angustifrons* is *M. levida* in the family Muscidae. As representatives of the family Muscidae, *M. angustifrons*, *M. levida* and *M. stabulans* was clustered in a single group, while *Haematobia irritans irritans* and *Musca domestica* showed another group even though they are representative of the family. This suggests the family Muscidae is a paraphyletic group. Similar result showed by the previous mitochondrial and nuclear data based research (Dsouli et al. 2011). Although Muscidae flies are important insects for the forensic entomology (Benecke 2001), it is hard to identify specimens by morphological examination because of highly similar morphological appearance according to metamorphic stages (Lan et al. 2015). Therefore, complete mitochondrial genome sequences can be important tool in forensic entomology for identifying species.

**Figure 1.**
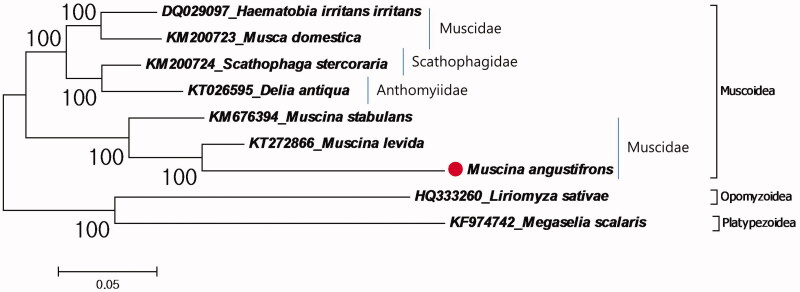
Molecular phylogeny of M. angustifrons in the superfamily Muscoidea. The complete mitochondrial genomes for reconstruction of phylogenetic tree retrieved from GenBank and the records belonging to the superfamily Opomyzoidea and Platypezoidea chosen as representative of outgroup. *M. angustifrons* record marked with a dot.
